# Reconstructive strategies using abdominal flaps for cutaneous malignancies: A case series

**DOI:** 10.1016/j.ijscr.2025.111301

**Published:** 2025-04-14

**Authors:** Aditya Sriharsha Pedaprolu, Suhas Jajoo, Bhushan Jajoo, Simran Dhole, Mehak Fayyaz Semy, Priya Chatterjee

**Affiliations:** a Department of General Surgery, Jawaharlal Nehru Institute of Medical College, Datta Meghe Institute of Higher Education and Research, Sawangi, (Meghe) Wardha, Maharashtra, India; b Department of General Medicine, DY Patil University, School of Medicine Nerul, Navi-Mumbai, Maharashtra, India; c Department of Pathology, Jawaharlal Nehru Institute of Medical College, Datta Meghe Institute of Higher Education and Research, Sawangi, (Meghe) Wardha, Maharashtra, India

**Keywords:** Abdominal flap reconstruction, Limb sparing surgery, Squamous cell carcinoma, Sarcoma, Case series, Plastic surgery

## Abstract

**Introduction and importance:**

Cutaneous lesions such as Marjolin's ulcer—a malignant transformation of chronic ulcerative wounds or scars into squamous cell carcinoma—and fungating soft tissue sarcomas, which are rapidly progressing tumours originating from mesenchymal tissues, exhibit a diverse range of clinical and pathological characteristics. These aggressive malignancies are characterized by high recurrence rates and poor prognoses. Traditionally, amputation was the primary treatment; however, limb salvage has become the preferred approach, necessitating extensive reconstruction with flap reconstruction.

**Case presentation:**

In this report, we present two cases of Marjolin's ulcer and one case of a fungating soft tissue sarcoma, detailing their clinical presentations, diagnostic workups, and management. Each patient underwent wide tumour excision followed by abdominal flap reconstruction.

**Clinical discussion:**

The shift from amputation to limb-sparing surgery highlights the advancements in oncologic and reconstructive techniques, allowing for better functional and aesthetic outcomes.

**Conclusion:**

This report underscores the critical role of early detection and a multidisciplinary approach in optimizing limb preservation and functional outcomes.

## Highlights

• We present three cases of three-dimensional wide excision of upper limb carcinomas with abdominal flap reconstruction.
• All patients recovered well post-operatively, with no evidence of recurrence or major flap complications during follow-up.
• This is achievable through thorough radiological and pathological investigations, combined with multidisciplinary management.
• Abdominal flap is a simple and reliable reconstructive option for managing upper limb defects following oncologic resection.

## 1 Introduction

Reconstruction using abdominal flaps has been a well-established procedure for many years and has gained traction with the advent of microsurgery and the use of free flaps. Adequate reconstruction is crucial for prioritizing limb-sparing surgery, the gold standard treatment for cutaneous malignancies, which we have highlighted with the use of abdominal flaps. These flaps are still employed for hand coverage in the current era of microsurgery [1,2].

Tumours such as squamous cell carcinoma (SCC) and fungating soft tissue sarcoma (FSTS) affect the epidermis and its appendages, and mesenchymal tissues, respectively [3,4]. Management involves a detailed history to identify risk factors, as well as clinical examination and histopathological diagnosis by taking a biopsy of the tumour or of its margins. We present three cases admitted in our rural medical centre: the first two involve patients diagnosed with SCC following a burn injury at the lesion site, while the third case concerns a patient diagnosed with FSTS. We analyze their clinical presentations, the investigations leading to diagnosis, and the subsequent multidisciplinary management aimed at salvaging the limb and maintaining function. Our case series has been reported in line with the PROCESS guidelines [5].

## 2 Case presentation

### 2.1 Case 1

A male patient in his 40s presented to our hospital with an ulcero-proliferative lesion on his right forearm, which had been present and causing discomfort for the past 6 months, rendering him unable to carry out his daily activities (Grade 1 on the ECOG performance status scale). A detailed history revealed a burn injury at the same location 12 years prior.

**Fig. 1:**
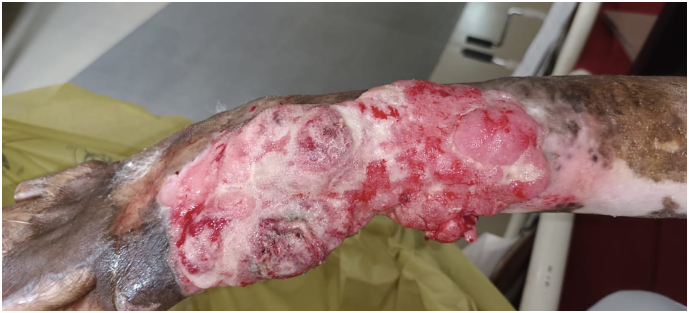
A pre-operative clinical Image of the wound.

Clinical examination revealed a 12 cm × 10 cm ulcerated, keratotic chronic wound on the right forearm with hypopigmented areas around the wound, exhibiting features that raised suspicion for malignant change (Fig. 1). Further local examination also revealed burn contractures on the hand and digits. A biopsy confirmed the diagnosis, showing histopathological evidence of well-differentiated squamous cell carcinoma. Our examination also revealed palpable lymph nodes in the right axilla, but cytopathology of these lymph nodes was negative for malignant cell infiltration.

**Fig. 2:**
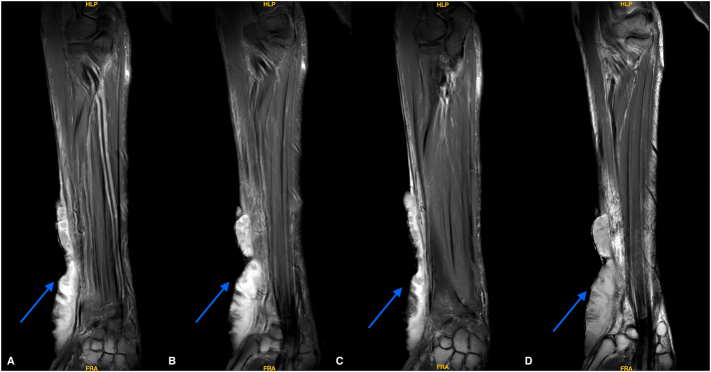
MRI images taken in Coronal T2 weighted (A and B) and T1 with Contrast images (C and D) with the tumour marked in Blue arrow. (For interpretation of the references to colour in this figure legend, the reader is referred to the web version of this article.)

Magnetic resonance imaging (MRI) indicated a large lobulated infiltrating soft tissue mass on the radial aspect of the distal forearm, involving the skin and subcutaneous tissue and diffusely abutting the deep fascia of the lateral and posterolateral aspects of the forearm, suggestive of early infiltration. Notably, there was no infiltration into muscle and bone (Fig. 2). A computed tomography (CT) scan of the abdomen and thorax showed no evidence of distant metastasis.

**Fig. 3:**
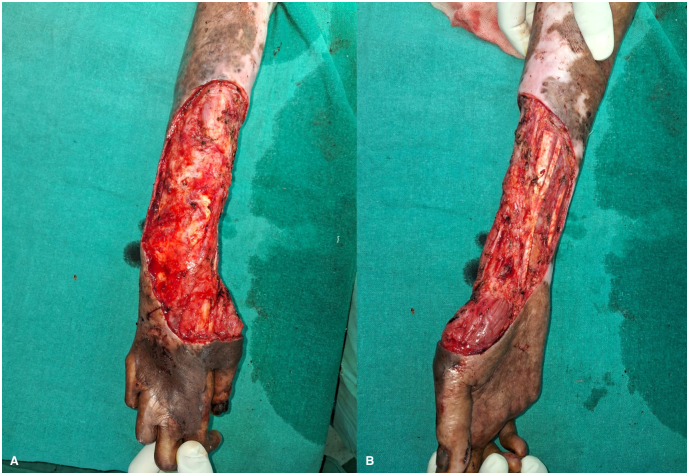
Intra-operative images (A and B) post wide local excision.

**Fig. 4:**
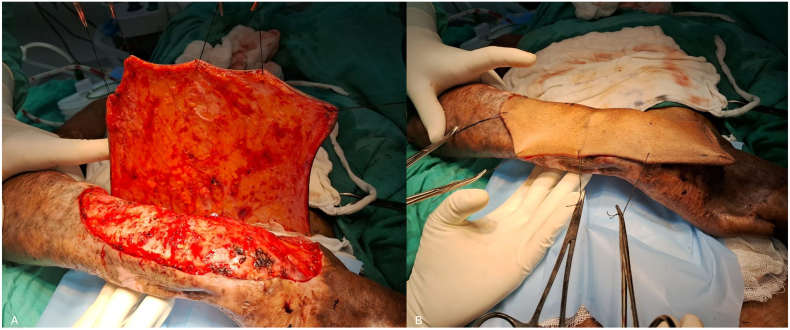
Images A and B depict Post-reconstruction intraoperative placement of abdominal flap.

**Fig. 5:**
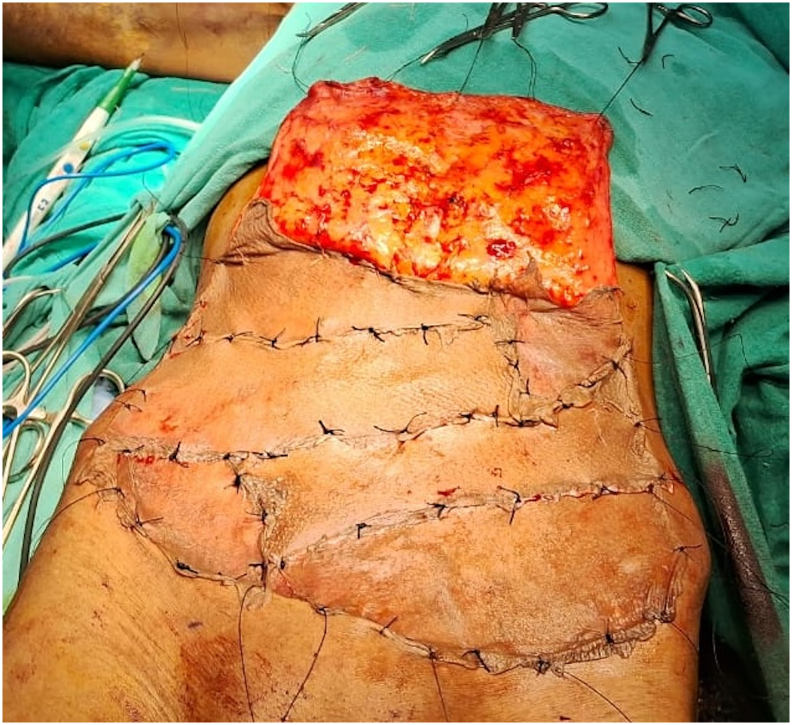
Intra-operative images post-abdominal flap harvest and split skin graft over harvest site.

Following a multidisciplinary discussion involving oncologists, plastic surgeons, and dermatologists, and considering the extensive nature of the disease, the patient was presented with two treatment options: amputation or limb salvage surgery. He was informed of the benefits, risks, and complications associated with each option. The patient opted for limb salvage surgery and was scheduled for a three-dimensional wide local excision of the tumour with a 1 cm margin (Fig. 3). We measured the dimensions of the defect and planned for reconstruction using a 15 × 15 cm random pattern abdominal flap, along with axillary lymph node dissection. The depth of the flap extended to Scarpa's fascia. Reconstruction was performed by placing the flap over the defect with non-absorbable interrupted sutures (Fig. 4), while the donor site was covered with a split-thickness skin graft harvested from the thigh region (Fig. 5).

**Fig. 6:**
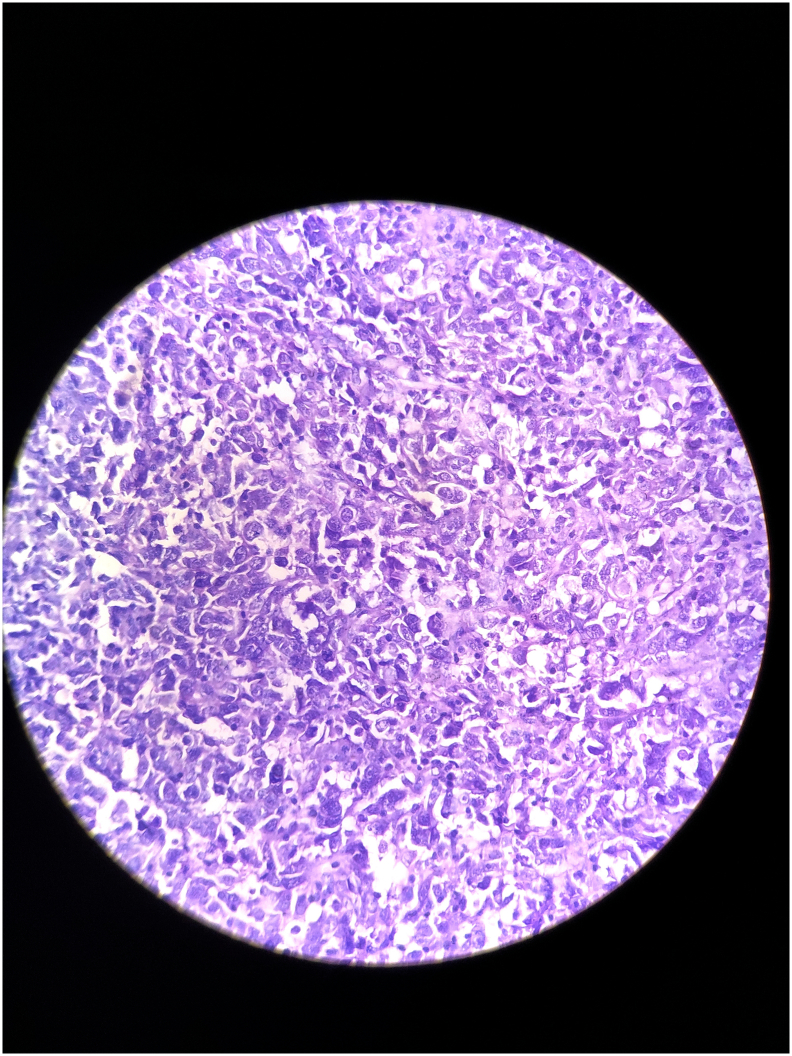
Section stained in H&E shows cells arranged in sheets. Tumour cells are large and pleomorphic. Nucleus is hyperchromatic showing high nucleus: cytoplasmic ratio with marked nuclear atypia and prominent nucleoli. Cytoplasm is eosinophilic. Keratinization is minimal.

Postoperative histopathology suggested poorly differentiated squamous cell carcinoma (Fig. 6), with margins being negative for malignant cell infiltration. Nine axillary lymph nodes were excised, and histopathological analysis indicated no signs of malignant cell infiltration.

**Fig. 7:**
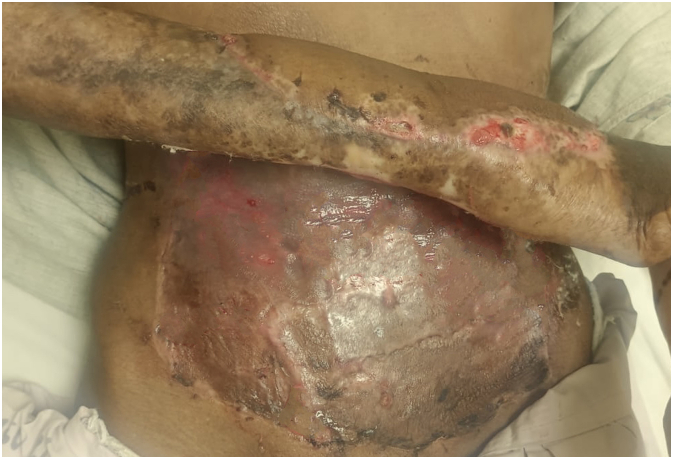
Wound follow-up on postoperative day 12.

The postoperative course was uneventful, with both the donor site and flap being in good condition (Fig. 7). He was discharged on postoperative day 7 with instructions to initiate passive range-of-motion exercises following consultation with our physiotherapist. Additionally, the patient was advised on appropriate local wound care and to maintain the upper limb in a functional position. Weekly follow-ups were conducted, and no wound or donor site complications were noted. Following flap division after 3 weeks, the patient was monitored with regular monthly follow-ups for 18 months. During this period, no delayed complications occurred, nor was there any evidence of recurrence of carcinoma and the patient achieved a satisfactory functional restoration.

### 2.2 Case 2

**Fig. 8:**
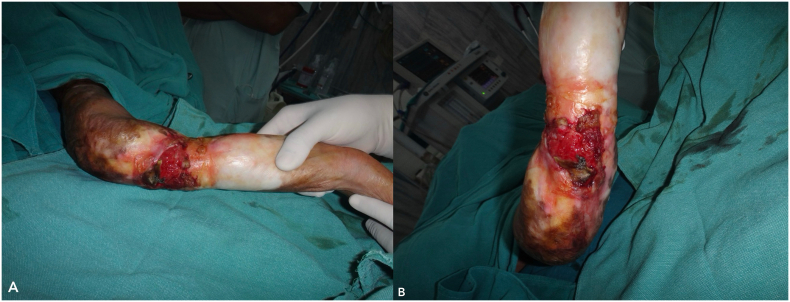
Pre-operative clinical Images (A and B) of the wound.

A female patient in her 50s presented to our hospital with complaints of an ulcero-proliferative lesion on her left forearm that had persisted for the past 6 months. Due to the growth of the ulcer, the patient was unable to carry out daily activities (Grade 1 on the ECOG performance status scale). She also had a history of a burn injury at the same site 18 years ago. Clinical examination revealed a 6 cm × 5 cm area of ulcerated, keratotic, chronic wound on the dorsal aspect of her left forearm (Fig. 8). There was no evidence of any axillary lymphadenopathy and a fine needle aspiration and cytology from the lymph node was negative for any malignant infiltration. A marginal biopsy of the wound suggested squamous cell carcinoma.

**Fig. 9:**
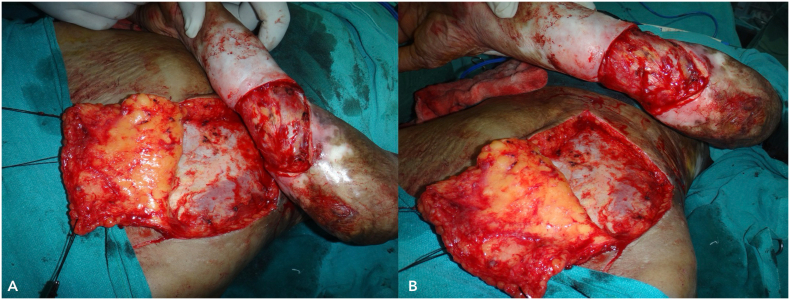
Intra-operative images (A and B) of Abdominal flap reconstruction and wide local excision.

A radiological examination, including a CT scan of the thorax and an MRI of her left forearm, was performed. The results showed no infiltration of the lesion into muscle or bone and no evidence of any distant metastasis. After thorough counselling and discussions involving various specialists, the patient opted for limb salvage surgery. She was scheduled for a three-dimensional wide local excision of the tumour. Malignant tissue along with adequate margins along with burn scars were excised. The dimensions of the defect were measured and we took a 12 × 9 cm random pattern abdominal flap over the quadrant of abdomen (Fig. 9). Since the lesion did not extend beyond the scar margins and the axillary lymph nodes were negative, we did not perform an axillary lymph node dissection. Reconstruction was performed by placing the flap over the defect with non-absorbable interrupted sutures and split thickness skin graft was placed over the donor site. Following the inset of the flap, the affected limb was immobilized. Histopathology report of the patient suggested well differentiated squamous cell carcinoma.

**Fig. 10:**
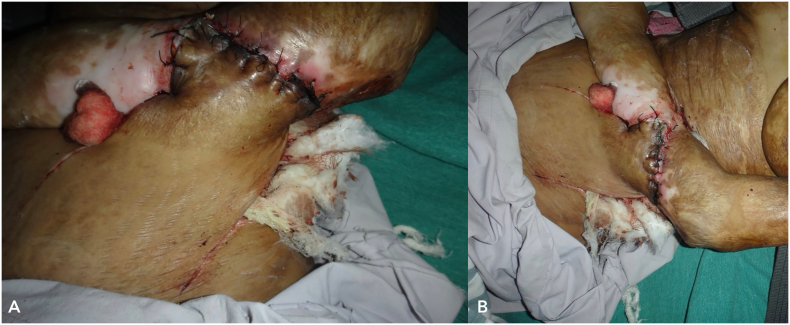
Post-operative images (A and B) following abdominal flap reconstruction.

Her postoperative course was uneventful, with thorough monitoring of both the donor site and flap for changes in skin colour, temperature, turgor, and capillary refill (Fig. 10). She was discharged on postoperative day 7 with similar instructions to begin passive range-of-motion exercises following consultation with our physiotherapist, along with local wound care and maintaining the upper limb in a functional position. Following flap division after 3 weeks, she was monitored with regular monthly follow-ups for 18 months. Throughout this period, no delayed complications were observed, nor was there any evidence of recurrence and the patient achieved a successful functional restoration.

### 2.3 Case 3

**Fig. 11:**
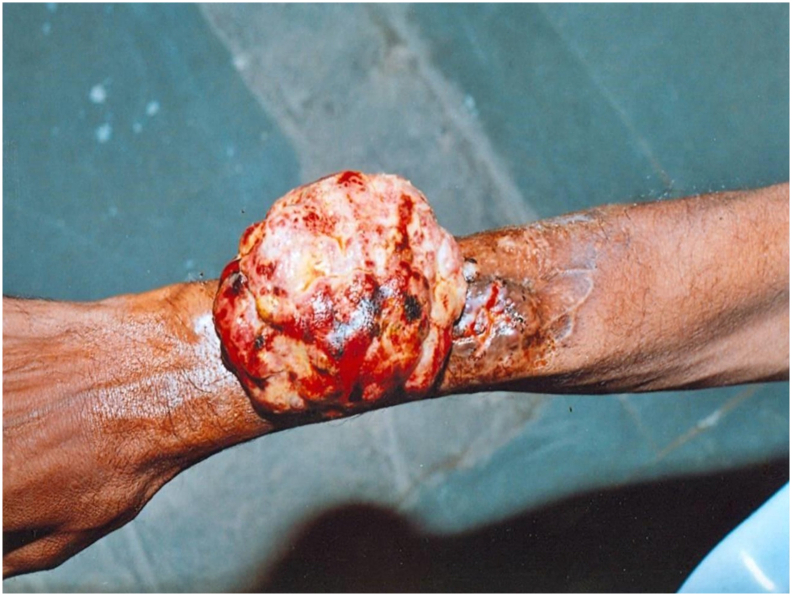
Pre-operative clinical Image of wound showing the ulcero-proliferative lesion.

A male patient in his 40s was referred to our hospital due to a rapidly progressive, ulcero-proliferative lesion on his right forearm, present for one year (Fig. 11). He reported a foul-smelling discharge from the swelling for the past two months. As the tumour progressed, it became fungated, rendering the patient unable to carry out any work activities (Grade 2 on the ECOG performance status scale). The patient, a farmer by profession, had no known comorbidities. Clinical examination revealed a 6 cm × 6 cm area of a chronic ulcerated, fungating wound on the dorsal aspect of his right forearm. There was no evidence of axillary lymphadenopathy and a cytology from the axillary lymph node was negative for malignant infiltration. A biopsy from the wound suggested fungating sarcoma.

**Fig. 12:**
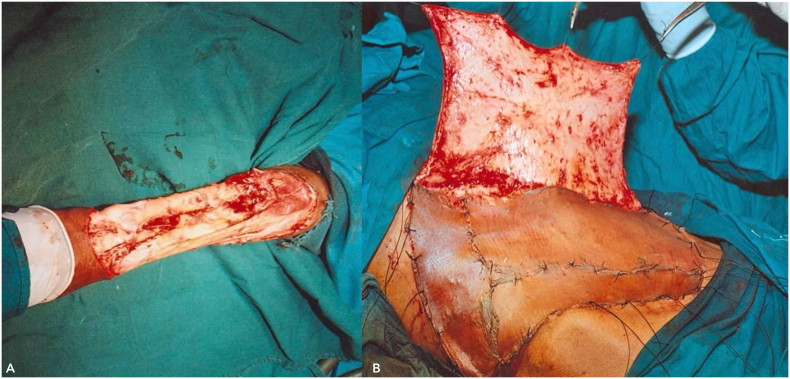
Intra-operative images (A and B) following wide local excision with abdominal flap reconstruction.

A radiological examination, including a CT scan of the thorax and an MRI of her left forearm, was performed. The results showed no infiltration of the lesion into muscle or bone and no evidence of any distant metastasis. After a multidisciplinary discussion, the patient was informed about the nature of his disease and counselled regarding its rapid progression and high likelihood of recurrence after surgery. We presented options for amputation and limb salvage surgery and explained the complications associated with each modality. Ultimately, the patient chose to preserve his limb and underwent a three-dimensional wide excision of the tumour. Complete excision of the malignant tissue with adequate margins was performed (Fig. 12A). The dimensions of the defect were measured, and reconstruction was achieved by covering the area with a 15 × 15 cm random pattern abdominal flap secured with non-absorbable interrupted sutures. The donor site was then covered with a split-thickness skin graft harvested from the thigh region (Fig. 12B).

**Fig. 13:**
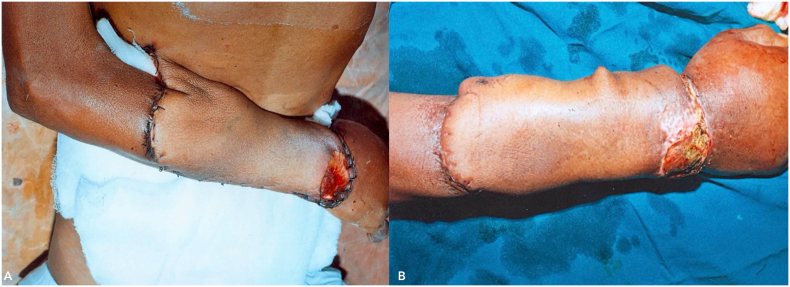
Post operative image following surgery on Post operative Day 5 (A) and following flap division on Post operative day 21.

**Fig. 14:**
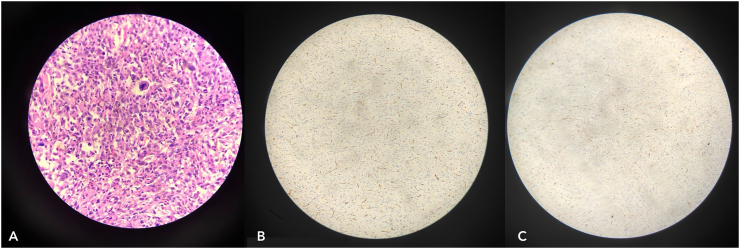
Section stained in H&E (A) shows cells arranged in sheets. Cells are showing significant pleomorphism. Nucleus are spindled showing hyperchromasia. Cytoplasm is eosinophilic. Mitosis is Brisk with some atypical forms. Bizzare giant cells are noted. Stroma is myxoid at places. IHC (B) CD34 and S100 (C) shows strong positivity.

The histopathology report indicated features consistent with fungating spindle cell carcinoma (FSCC), with positive margins for infiltration. IHC (CD-34 and S100) analysis showed strong positivity that confirmed our diagnosis of FSCC (Fig. 13). On post-operative day 3, he developed a minor flap loss along the margins, for which he was reassured that the wound would heal by secondary intention (Fig. 14). Apart from this minor difficulty, he did not develop any other wound complications. Following flap division after 3 weeks, he was discharged with advice to begin range of motion exercises and was referred to the oncology department after a multidisciplinary consultation for adjuvant chemo-radiation.

## 3 Discussion

Marjolin's ulcer (MU) was first described by Jean Nicholas Marjolin in 1828 and reported by Hawkins in 1835 in a case of squamous cell carcinoma that appeared to originate from a site of chronic osteomyelitis. Based on the duration, MUs are classified as acute (less than 1 year) or chronic (more than 1 year). Squamous cell carcinoma (SCC) is characterized by malignant cells that demonstrate maturation towards keratin formation. This tumour arises from the stratum basalis layer of the epidermis and is the second most common cutaneous carcinoma after basal cell carcinoma (also known as rodent ulcer) [[6], [7], [8]].

Risk factors for SCC include prolonged exposure to ultraviolet radiation (primarily UV-B rays), ionizing radiation following radiotherapy, exposure to chemicals such as arsenic, coal tar, and hydrocarbons, and immunosuppression (with a 100-fold increased risk following organ transplantation). Additional risk factors encompass human papillomavirus (HPV) infection, chronic inflammation associated with pre-existing post-traumatic scars and chronic wounds (including Marjolin's ulcer), chronic sinus tracts, chronic osteomyelitis, previous vaccination sites, and burn injuries, as seen in our case. Early detection of precursor lesions such as cutaneous horn, actinic keratosis, keratoacanthoma, or Bowen's disease is crucial. SCC is also linked to genetic skin cancer syndromes like xeroderma pigmentosum, albinism, Muir-Torre syndrome, dystrophic epidermolysis bullosa, and Fanconi anaemia. Anatomically, 80 % of SCC cases involve the head and neck, while only 5 % involve the upper limbs. However, SCC consistent with Marjolin's ulcer is uncommon, with an incidence ranging from 0.23 % to 1.7 %.

There should always be a degree of suspicion for malignancy in chronic wounds exhibiting certain characteristic changes. These changes include a foul-smelling, enlarging, or exophytic mass accompanied by increased pain, lymphadenopathy, or bleeding. A lack of response to treatment is concerning, while progressive bone destruction seen on radiographs or a persistent lesion with an enlarged mass indicates invasive disease [9]. Squamous cell carcinoma (SCC) has a higher metastatic potential than basal cell carcinoma. The most common site for metastasis is the regional lymph nodes, with distant metastases typically involving the underlying bone, brain, and lungs. In cases of head and neck cancers, the parotid gland is also a significant area of concern. Management of SCC varies based on the disease's nature, prognosis, and staging. The goal of treating Marjolin's ulcer is complete excision of the tumour, which necessitates thorough evaluation using CT or MRI, along with tumour and lymph node biopsies. While wide local excision is the preferred method for tumour removal to ensure clear margins, techniques such as Mohs micrographic surgery or curettage with electro-dissection may be employed in select cases [10,11].

Soft tissue sarcomas (STS), which account for less than 1 % of all newly diagnosed malignancies, commonly occur in the extremities. These tumours typically present as painless, progressively enlarging masses. Forearm sarcomas are exceedingly rare and frequently mistaken for benign soft tissue tumours [12]. Cutaneous involvement from a soft tissue sarcoma is rare and usually observed in patients with primary carcinomas, such as breast cancer or melanoma. This tumour can erode through the dermis, creating a connection with the surface—a process known as malignant ulceration or fungation [13]. Diagnosis is essential, as differential diagnoses can include basal cell carcinoma, liposarcoma, or local inflammatory conditions such as an abscess. Investigations to determine the extent of the condition can be conducted through clinical and radiological examination of the affected area. While MRI is the preferred method of investigation, financial constraints often make it difficult to perform these assessments.

Surgical resection is the cornerstone of STS treatment and is often followed by adjuvant radio- or chemotherapy. The goal of curative surgery is to achieve tumour-free margins within an acceptable resection range, typically involving a 10-mm surgical margin. Historically, amputation was considered the gold standard for treating STS; however, numerous studies now show that amputation does not improve overall survival rates in patients eligible for limb-sparing surgery. Therefore, it is crucial to initiate treatment early to prevent extensive invasion, as limb amputation—although an accepted treatment alongside local excision—can lead to significant complications and adversely affect a patient's quality of life. This is particularly important for cutaneous SCC and FSTS, where early detection can help avoid many amputations [14].

Full-thickness skin grafts (FTSGs) can serve as an alternative to flap reconstruction. However, FTSGs carry a greater risk of graft failure, as their survival depends on the ingrowth of capillaries from the recipient site. FTSGs should not be placed in defects of any size without proper underlying blood supply. Large areas of exposed bone, cartilage, or tendons/ligaments are also not optimal for graft placement, as observed in our cases. There are also donor site complications that must be taken into account, as they must be closed or undergo a longer healing period by secondary intention. Cosmetically, harmonizing the graft with the surrounding colour and texture can also be challenging. Hence, after considering these options and conferring with our patients, we opted for flap reconstruction, believing we could achieve a better cosmetic and functional effect by covering a large defect and achieving a higher survival rate than a skin graft [15,16].

Abdominal flaps,which are bulky and versatile, are our preferred choice in reconstructive surgery due to their reliability, especially in patients with comorbidities who cannot undergo lengthy and complex procedures. There are four main types of abdominal flaps: the groin flap, which is based on the Superficial Circumflex Iliac Artery (SCIA) and is the most commonly used; the Superficial External Pudendal Artery (SEPA) flap, supplied by the corresponding artery; the Superficial Inferior Epigastric Artery (SIEA) flap; and the paraumbilical perforators (PUP) flap, which is supplied by perforators of the deep inferior epigastric artery [17,18]. In case of random pattern skin flaps, the abdominal skin and subcutaneous tissue taken are based on unnamed vessels [19]. This flap reconstruction is an excellent option for patients with cutaneous malignancies, as these flaps tolerate adjuvant therapies better than grafts. A key disadvantage of local flaps is the need for a two-stage procedure and the potential for postoperative complications such as hypertrophic scarring, keloid formation, limb stiffness, reduced limb strength, and sensory loss. These issues can be mitigated through prolonged rehabilitation, starting with passive and active range-of-motion exercises alongside the physiotherapist, followed by active physiotherapy after one month. During this period, keeping the upper limb in a functional position and providing proper wound care encouraging the use of elastic compression or silicone sheet application. With the advancement of microvascular surgery, free flaps have become a viable option, offering significant advantages in terms of blood supply. A single-stage reconstruction, followed by early rehabilitation, can reduce the risk of adhesions and stiffness, leading to a quicker and more efficient recovery [20]. However, the complexity of microvascular procedures requires advanced microsurgical skills, which may result in a higher risk of failure.

Considering all these factors, we believe that the choice of surgical technique ultimately depends on the surgeon's experience, technical proficiency, patient preferences, and the resources available at different medical centres. The medical management of cutaneous malignancies may involve radiotherapy, which can be used as a primary treatment for inoperable tumours or as an adjunctive therapy. Systemic chemotherapy, such as a combination of cisplatin with 5-fluorouracil, carboplatin, or EGFR inhibitors, is typically employed as adjuvant therapy or for treating locally advanced, inoperable, or metastatic disease. Localized chemotherapy, using topical agents or cryotherapy, is primarily reserved for premalignant lesions [21,22].

## 4 Conclusion

Our case series demonstrates the effectiveness of abdominal flaps as a simple and reliable reconstructive option for managing upper limb defects following oncologic resection. These flaps can cover extensive defects without increasing patient morbidity. By using these techniques, we can prioritize limb salvage over amputation, thereby preserving function and enhancing postoperative recovery and functional restoration. Although microsurgical free flaps offer certain advantages, abdominal flaps remain a viable alternative that can be easily learned. They do not require rigid anatomical landmarks and can be performed in various settings, particularly in resource-limited environments or for patients with contraindications to complex microvascular procedures.

However, the management of cutaneous malignancies requires a multidisciplinary approach that incorporates oncologic resection, with or without adjuvant therapy, reconstructive surgery, and comprehensive physiotherapy and rehabilitation. This approach is essential for achieving optimal functional and aesthetic outcomes. Future research can focus on refining flap selection criteria, evaluating long-term patient-reported outcomes, and integrating advanced rehabilitation protocols to further enhance reconstructive strategies in oncologic limb preservation.

## Author contribution

ASP: Conceptualization, design of the study, acquisition of data, drafting the article, revising it critically for important intellectual content, approval of the version to be submitted.

SJ: Conceptualization, design of the study, acquisition of data, drafting the article, revising it critically for important intellectual content, approval of the version to be submitted.

BJ: Analysis, drafting the article, revising it critically for important intellectual content, approval of the version to be submitted.

SD: Acquisition of data, analysis, revising it critically for important intellectual content, approval of the version to be submitted.

MFS: Acquisition of data, analysis, revising it critically for important intellectual content, approval of the version to be submitted.

PC: Acquisition of data, analysis, revising it critically for important intellectual content, approval of the version to be submitted.

## Consent

Written informed consent was obtained from the patient for publication of this case report and accompanying images. A copy of the written consent is available for review by Editor-In-Chief of this journal on request.

## Ethical approval

Ethical approval is not required in our institution (Institutional Ethics Committee of the Medical University of Datta Meghe Institute of Higher education and research) for case reports and case series.

## Guarantor

Aditya Sriharsha Pedaprolu is the Guarantor.

## Research registration number

Registration for research study is not applicable.

## Funding

This research did not receive any financial support.

## Conflict of interest statement

The authors declare no conflicts of interest.
